# Molecular detection of Rickettsiales and a potential novel *Ehrlichia* species closely related to *Ehrlichia chaffeensis* in ticks (Acari: Ixodidae) from Shaanxi Province, China, in 2022 to 2023

**DOI:** 10.3389/fmicb.2023.1331434

**Published:** 2024-01-11

**Authors:** Xue Zhang, Wen Lv, Zhongqiu Teng, Na Zhao, Yue Zhou, Di Ma, Lin Ma, Yuqing Cheng, Jianjun Wei, Jia He, Wenke Ma, Dongli Liu, Tian Qin

**Affiliations:** ^1^National Key Laboratory of Intelligent Tracking and Forecasting for Infectious Diseases, National Institute for Communicable Disease Control and Prevention, Chinese Center for Disease Control and Prevention, Beijing, China; ^2^Shaanxi Provincial Center for Disease Control and Prevention, Xi'an, China; ^3^Long County Center for Disease Control and Prevention, Baoji, China; ^4^Mei County Center for Disease Control and Prevention, Baoji, China; ^5^HanZhong Center for Disease Control and Prevention, Hanzhong, China; ^6^Zhenba County Center for Disease Control and Prevention, Hanzhong, China

**Keywords:** SFGR, *Anaplasma*, *Ehrlichia*, Ixodidae, Shaanxi Province

## Abstract

Important tick-borne diseases include spotted fever group *Rickettsia* (SFGR), *Anaplasma*, and *Ehrlichia*, which cause harm to animal and human health. Ixodidae are the primary vectors of these pathogens. We aimed to analyze the prevalence and genetic diversity of SFGR, Anaplasma, and *Ehrlichia* species in the Ixodidae in Shaanxi Province, China. Herein, 1,113 adult Ixodidae ticks were collected from domestic cattle and goats, and detected using nested PCR. A total of four Ixodidae species were collected and *Ca.* R. jingxinensis (20.58%, 229/1113), *A. bovis* (3.05%, 34/1113), *A. capra* (3.32%, 37/1113), *A. marginale* (0.18%, 2/1113), *E.* sp. Yonaguni138 (0.18%, 2/1113), and a potent novel *Ehrlichia* species named *E.* sp. Baoji96 (0.09%, 1/1113) were detected. *A. marginale* was detected for the first time in *Rhipicephalus microplus*. *E*. sp. Baoji96 was closely related to *E. chaffeensis* and was first identified in *Haemaphysalis longicornis*. In addition, co-infection with two Rickettsiales pathogens within an individual tick was detected in 10 (1.54%) ticks. This study provides a reference for the formulation of biological control strategies for ticks and tick-borne diseases in Shaanxi Province, and could lead to an improved control effect.

## 1 Introduction

Rickettsiales are a class of bacteria that are gram-negative obligate intracellular bacteria that can infect humans and different vertebrates via the bite of an arthropod vector. Recognized important Rickettsiales pathogens include spotted fever group *Rickettsia* (SFGR), *Anaplasma*, and *Ehrlichia*, and Ixodidae are their primary vectors (Qin et al., 2019). The initial symptoms are similar to those of a cold, such as high fever, weakness, pain, chills, etc., and there are also signature clinical features, rash and eschar. SFGR consists of over 30 species distributed worldwide, and at least 18 species have been identified as human pathogens in China (Robinson et al., 2019; Teng et al., 2023b). For example, *Rickettsia rickettsii* is the most pathogenicSFGR, which causes Rocky Mountain spotted fever (RMSF) in North America. *Rickettsia conorii* causes Mediterranean spotted fever (MSF) in some regions of Europe, Africa, and Asia (Robinson et al., 2019). In addition, disease names associated with pathogens often reflect the region where they are found; however, the actual endemic region is often much larger (Labruna et al., 2014). *Rickettsia japonica* causes Japanese spotted fever (JSF). It was first described in Japan in 1984 in three patients with high fever and rash (Mahara, 1997). But outside Japan, cases have been reported in China, South Korea, the Philippines, and Thailand (Teng et al., 2023a). At present, the genera *Anaplasma* and *Ehrlichia* contain eight known bacterial species that infect humans: *A. phagocytophilum*, *A. platys*, *A. ovis*, *A. bovis*, *A. capra*, *E. chaffeensis*, *E. ewingii*, and *E. muris* (Bakken et al., 1994; Paddock and Childs, 2003; Thomas et al., 2009; Chochlakis et al., 2010; Johnson et al., 2015; Li et al., 2015; Lu et al., 2022). Furthermore, *E. chaffeensis* causes human monocytotropic ehrlichiosis (HME), and *A. phagocytophilum* is responsible for human granulocytotropic anaplasmosis (HGA). Both HME and HGA have fairly high infection rates in the United States, and cases have also been reported in China (Ismail et al., 2010). Rickettsiales are potential pathogenic factors for numerous diseases, which can lead to rash, post-infectious arthritides, interstitial pneumonia, meningoencephalitis, acute kidney injury, multiple organ failure, and even death after human infection (Pasquale Mansueto et al., 2012; Zeidler and Hudson, 2021; Sebastian et al., 2022). Moreover, in recent years, a number of emerging and re-emerging Rickettsiales species have been discovered to infect humans (Qin et al., 2019). Thus, Rickettsiales diseases will continue to be a threat to human health.

The epidemiology of tick-borne Rickettsial disease reflects the geographic and seasonal distribution of the pathogen and transmission is mainly related to the following: the tick vector and its host, and the human behavior of people bitten after their skin is exposed to the tick (Beati et al., 1997). The distribution of tick-borne Rickettsial disease is geographically similar to that of ticks and their hosts, which tells us that understanding the distribution and changes of vectors is very important for the prevention and control of tick-borne rickettsial disease. Ticks are found in a wide range of areas, ranging from forests to roadside bushes, and even in areas without vegetation (Piotrowski and Rymaszewska, 2020). This makes us aware of the potential dangers of tick bites when we travel.

Ixodidae, the primary vectors of pathogens belonging to the Rickettsiales, comprise 111 species from 7 genera in China (Zhang et al., 2019). Ticks are found in all regions of China and infect every class of terrestrial vertebrates, including mammals, birds, reptiles, and even amphibians (Lu et al., 2019). With more and more attention being paid to the construction of urban landscaping, the contact area between man and nature has been greatly increased. This overlaps human, animal and tick habitats, greatly increasing the risk of human tick bites. Despite their ubiquity in nature, these organisms are often overlooked as an important cause of disease around the world. Treatment is easily delayed because of a lack of awareness of tick-borne diseases. Due to misdiagnosis, the best antibiotic treatment time is missed, which may lead to serious complications and even death. Hence, investigation of local tick-borne Rickettsiales pathogen prevalence is helpful for the early diagnosis and treatment of related diseases.

The terrain of Shaanxi province consists of mountains, plains, and basins, spanning three climatic zones. Southern Shaanxi is humid, central Guanzhong is semi-humid, and northern Shaanxi is semi-arid; therefore, it is rich in species and diverse in vegetation types. The Qinling Mountains are even known as a “biological gene bank.” Different ticks have different habitats. *Ixodes* prefer cool, moist environments, but *Rhipicephalus sanguineus* are adapted to high temperatures and dry environments. And, Different ticks prefer different hosts (Piotrowski and Rymaszewska, 2020). Here, ticks always find the right living conditions and plenty of hosts. In the past, Rickettsiales studies in Shaanxi Province mainly focused on *Anaplasma*, especially *A. capra* (Guo et al., 2019b). In this study, we aimed to analyze the prevalence and genetic diversity of SFGR, *Anaplasma*, and *Ehrlichia* species in Ixodidae collected from Shaanxi Province, China.

## 2 Materials and methods

### 2.1 Sample collection

**Figure 1:**
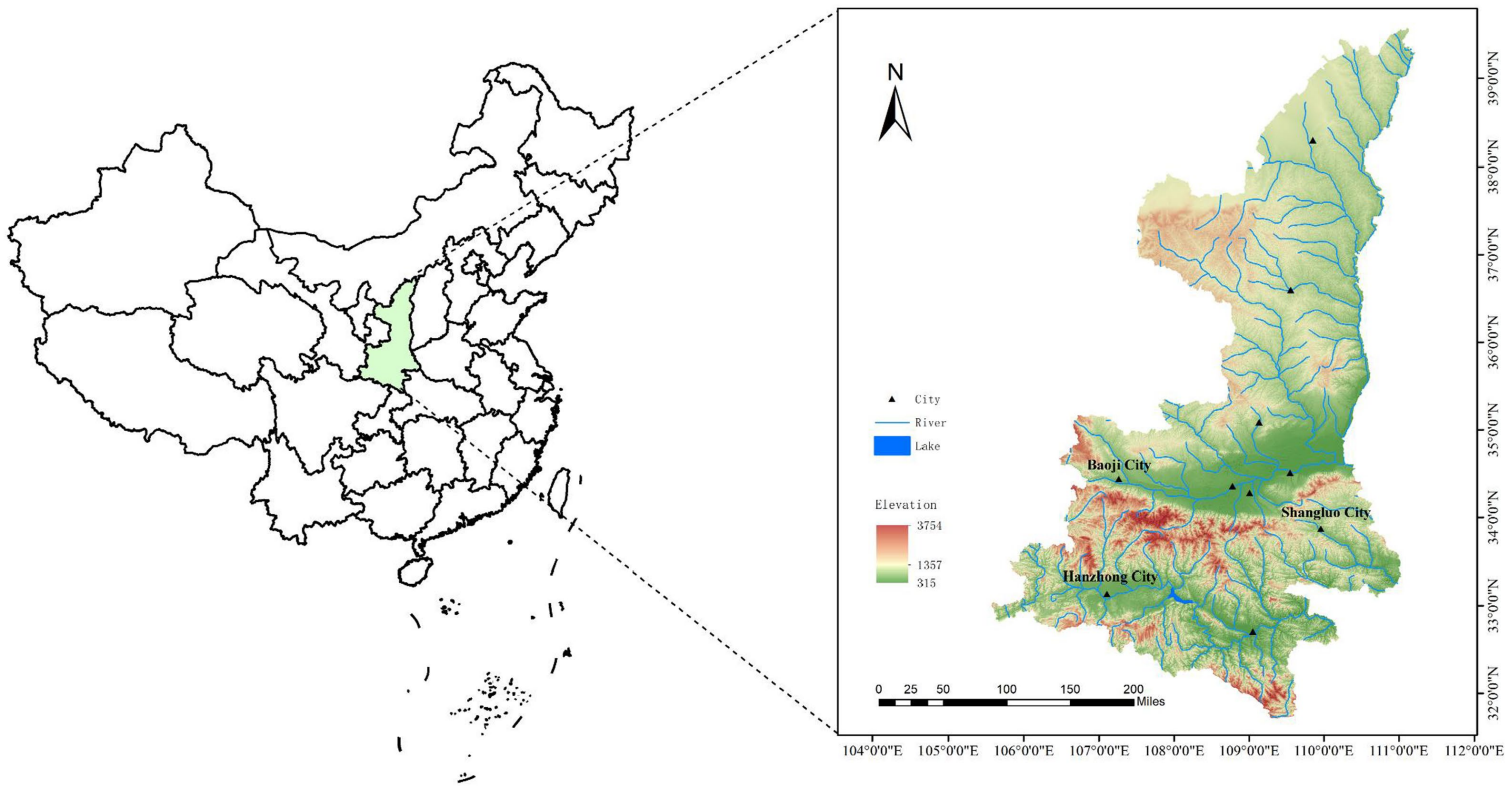
Map of Shaanxi Province showing the locations of the study sites.

From 2022 to 2023, adult ticks were collected from locations in Shaanxi Province, China: Zhenba County of Hanzhong city (32°08′ ~ 32°50′N, 107°25′ ~ 108°16′E), Baoji City (33°35′ ~ 35°06′N, 106°18′ ~ 108°03′E), and Shangluo District of Shangluo City (33°06′ ~ 33°44′N, 10°24′ ~ 111°01′E) (Figure 1). Two methods of collecting ticks were used: the tweezers were used to pick up ticks on the body surface of domestic animals (cattle and goats), and the cloth flag method was used to collect ticks in grassland. All ticks were identified by observing their structure under a light microscope and referring to the tick classification search table (Teng and Jiang, 1991).

### 2.2 DNA extraction

The ticks were removed from −80°C storage, and washed successively using 0.1% Bromo-Germaine, 75% alcohol, and phosphate-buffered saline (PBS) for 10–15 min. This step is to remove impurities from the surface of ticks. The ticks were individually homogenized in PBS, and then centrifuged for 3 min at 2,500 × g. Total nucleic acids from the tick homogenates were extracted using a QIAamp DNA Mini Kit (Qiagen, Hilden, Germany) and diluted to 100 μL. All the DNA extracts were stored at −20°C until use.

### 2.3 PCR amplification and sequencing

All ticks were further confirmed by PCR amplification and DNA sequencing of the mitochondrial cytochrome oxidase I (*CO* I) gene (Peng-Fei et al., 2018). *Rickettsia* in the ticks were simultaneously detected using nested or semi-nested PCR targeting a 440 bp region of the 17-kDa antigen-encoding (17kD) gene, a 1,100 bp region of the citrate synthase (*glt*A) gene, and a 1,200 bp region of the 16S rRNA (*rrs*) gene. A 500 bp sequence generated using universal primers of the *rrs* gene was used to detect *Anaplasma* and *Ehrlichia*. The clones positive for *Anaplasma* were further amplified using primers amplifying *gro*EL (encoding the 60 kDa heat shock protein), *glt*A, and a longer fragment (1,200 bp) of the *rrs* gene. The clones positive for *Ehrlichia* were further amplified with primers for *gro*EL, *glt*A, *dsb* (encoding the disulfide bond formation protein), *fts*Z (encoding a cell division protein), and a longer fragment of the *rrs* gene. For the potential novel agents, positive specimens a 1,200 bp fragment of the *rrs* gene was amplified, and two nested PCR assays were used to amplify the 5′-end and 3′-end fragments of the *rrs* gene to assemble a complete gene (Wen et al., 2002). All the primers used for PCR are shown in Supplementary Table S1. All primers were synthesized by Sangon Biotech (Shanghai) Co., Ltd. And PCR was performed using Premix Taq Version 2.0 plus dye (Takara, Dalian, China). These sequences were amplified by nested or semi-nested PCR according to Lu et al. (2022). Tm is 50°C.

The PCR products were electrophoresed through 1.0% agarose gels. The target amplicons were purified using a QIAquick PCR Purification Kit (Qiagen). The purified PCR products were sent to Beijing De’aoping Biotechnology Co., Ltd. (Beijing, China) for bi-directional sequencing.

### 2.4 Genetic and phylogenetic analysis

The sequences obtained from the target genes were modified and assembled using the EditSeq and SeqMan programs (in DNAStar, Ver. 7.0, DNASTAR Inc., Madison, WI, United States) to make them accurate and complete. For confirmation, these sequences were compared with those uploaded to GenBank^1^ using the Basic Local Alignment Search Tool (BLASTn). Multiple sequence alignments of these sequences were performed using the Clustal W method (with default parameters) as implemented in the MegAlign program (DNAStar, Ver. 7.0). The evolutionary history of these sequences was inferred using the maximum likelihood method in MEGA version 7 (Bakken et al., 1994; Kumar et al., 2016). The robustness of the resultant phylogenetic trees was assessed based on bootstrap support values obtained from 1,000 replicates; values more than 70% were considered to indicate significant differences.

### 2.5 Nucleotide sequence accession number

The GenBank accession numbers of the nucleotide sequences obtained in this study are presented in Supplementary Table S2.

## 3 Results

### 3.1 Tick collection and identification

A total of 1,113 adult ticks were collected from three different locations in Shaanxi Province (365 from Zhenba County, 691 from Baoji City, 57 from Shangluo District) (Figure 1). Based on the tick classification characteristics and further confirmed by amplification of the *COI* gene, three tick species were identified: *Haemaphysalis longicornis* (87.42%, 973/1113), *Rhipicephalus microplus* (11.77%, 131/1113), *Haemaphysalis flava* (0.81%, 9/1113) (Table 1). The *COI* gene sequences of all the ticks obtained in this study showed 99–100% identities with those of the above three ticks in GenBank (OR574171–OR574179).

**Table 1:** Detection of *Rickettsia*, *Anaplasma*, and *Ehrlichia* in ticks from different areas in Shaanxi Province, China.

Locality			Ticks				
Province	County	Geographic coordinates	Species	No. tested	*Rickettsia* (No. of positive)	*Anaplasma* (No. of positive)	Ehrlichia (No. of positive)
Shaanxi	Zhenba	32°08′ ~ 32°50′N, 107°25′ ~ 108°16′E	*Haemaphysalis longicornis*	230	*Ca.* R. jingxinensis (29)	*A. bovis* (11)	*E.* sp. Yonaguni138 (2)
			*Rhipicephalus microplus*	131	*Ca.* R. jingxinensis (31)	*A. marginale* (2)	0
			*Haemaphysalis flava*	4	*Ca.* R. jingxinensis (3)	0	0
	Baoji	33°35′ ~ 35°06′N, 106°18′ ~ 108°03′E	*Haemaphysalis longicornis*	686	*Ca.* R. jingxinensis (165)	*A. bovis* (23)	*E.* sp. Baoji96 (1)
						*A. capra* (36)	
			*Haemaphysalis flava*	5	0	0	0
	Shangluo	33°06′ ~ 33°44′N, 110°24′ ~ 111°01′E	*Haemaphysalis longicornis*	57	*Ca.* R. jingxinensis (1)	*A. capra* (1)	0
Total				1,113	229 (20.58%)	73 (6.56%)	3 (0.27%)

### 3.2 Detection and phylogenic analysis of Rickettsia

**Figure 2:**
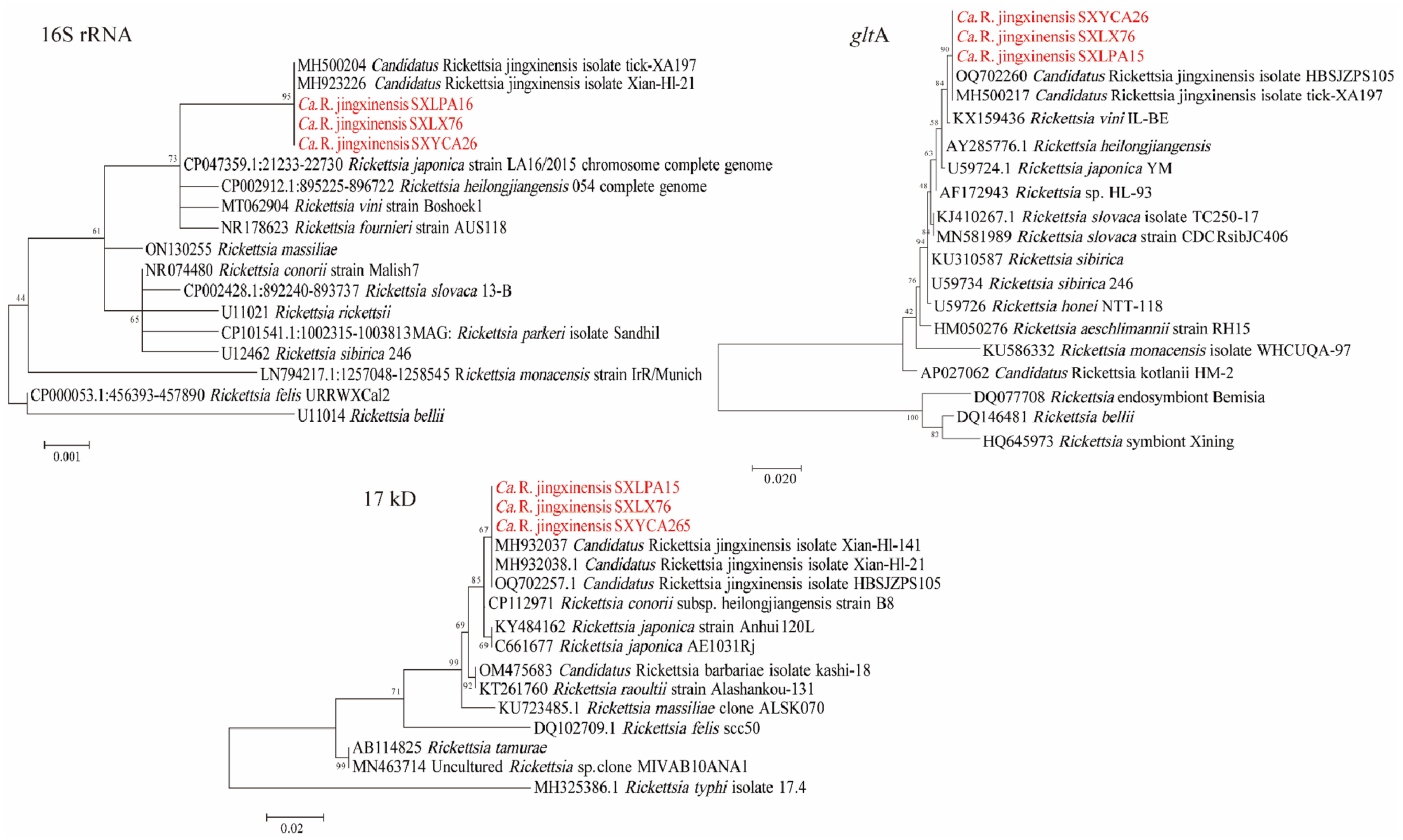
Phylogenetic trees of *Rickettsia* strains. The tree was constructed using MEGA 7.0 based on the nucleotide sequences of the 16S rRNA (1,188 bp), gltA (1,000 bp; encoding citrate synthase), and 17kD (440 bp, encoding the 17-kDa antigen) genes. Sequences obtained in this study are marked in red.

**Figure 3:**
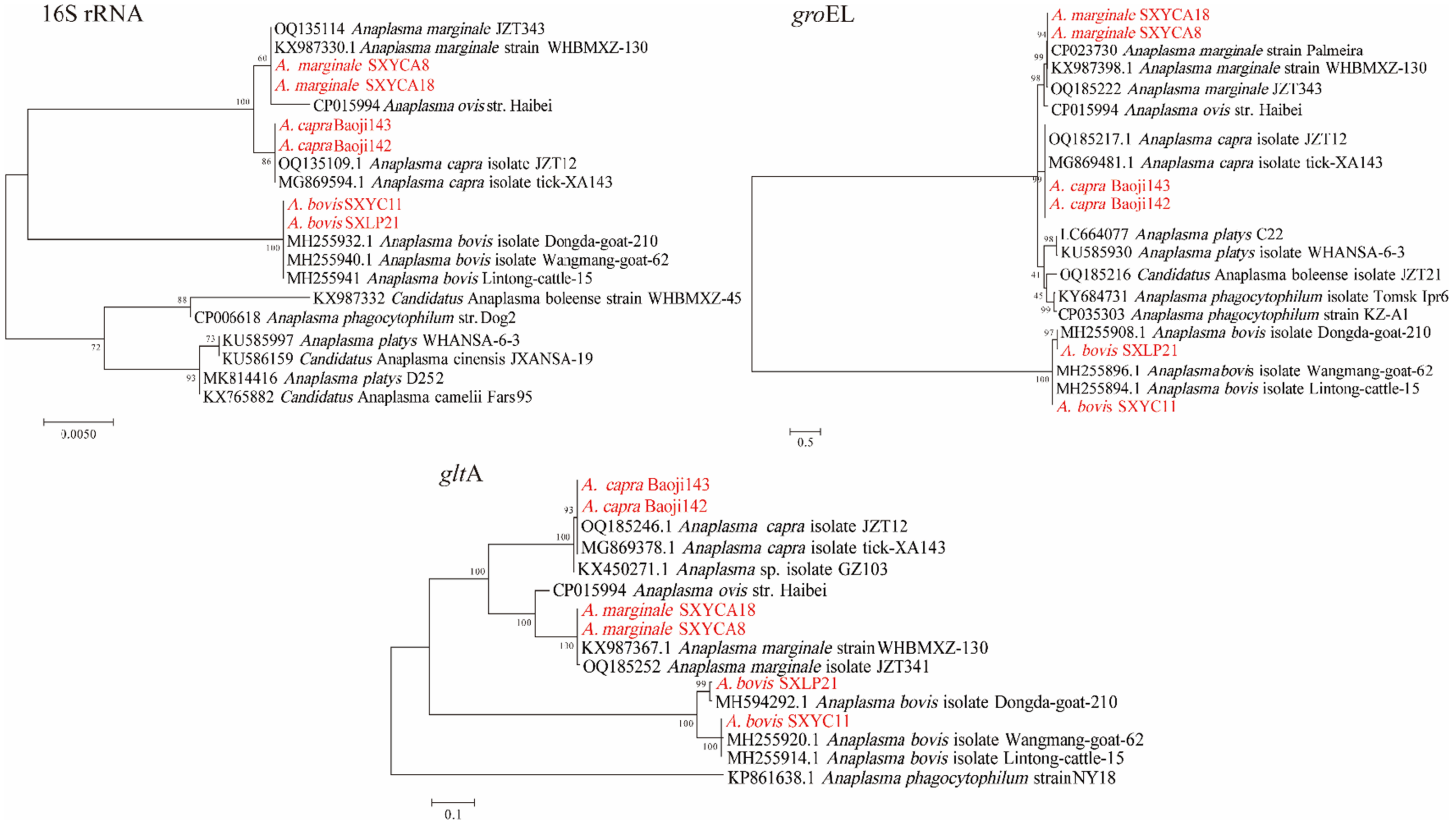
Phylogenetic trees of *Anaplasma* strains. The tree was constructed using MEGA 7.0 based on the nucleotide sequences of the 16S rRNA (1,200 bp), gltA (1,000 bp), and groEL (800 bp, encoding the 60 kDa heat shock protein) genes. Sequences obtained in this study are marked in red.

**Figure 4:**
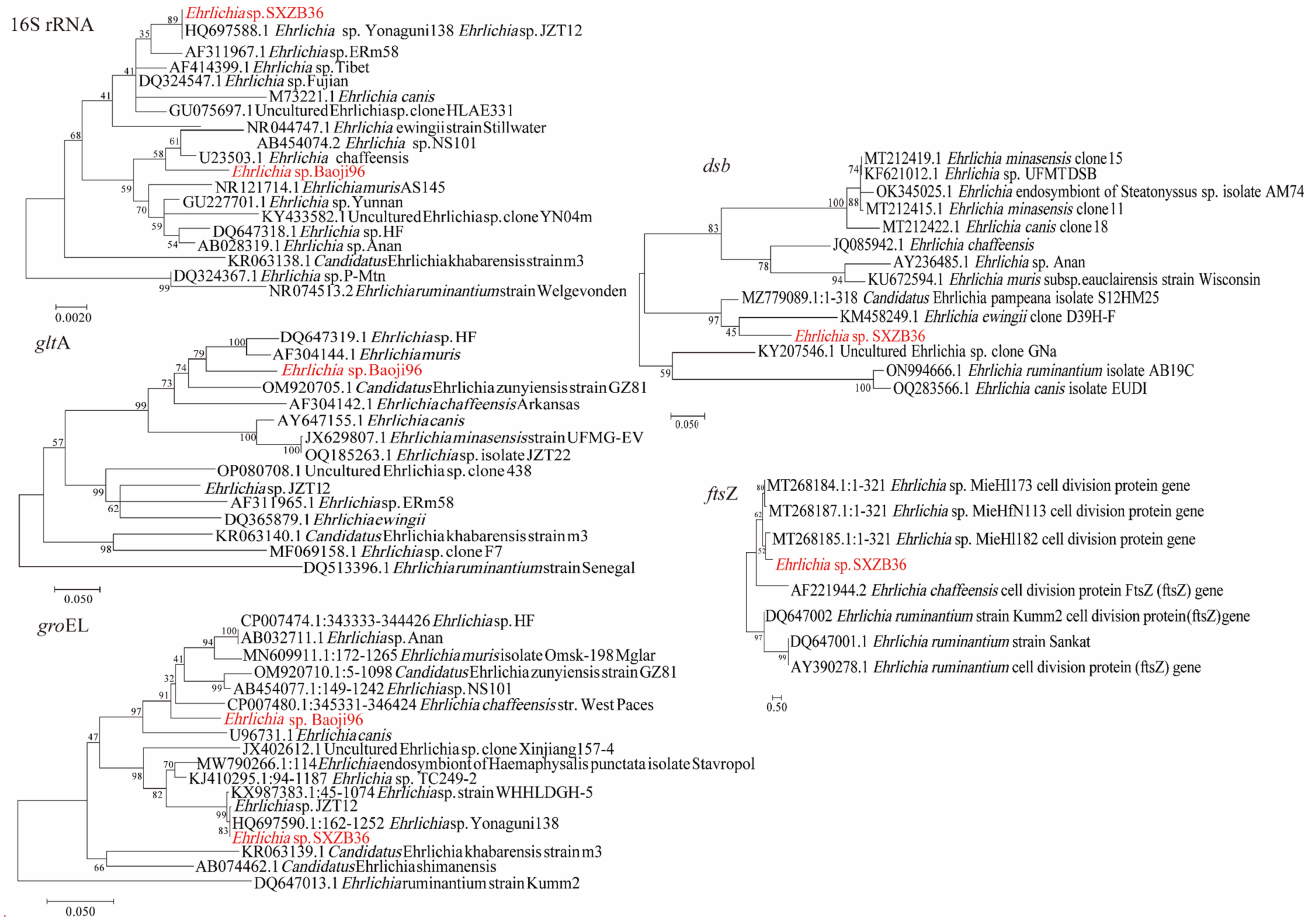
Phylogenetic trees of *Ehrlichia* strains. The tree was constructed using MEGA 7.0 based on the nucleotide sequences of the 16S rRNA (1,110 bp), gltA (804 bp), groEL (1,030 bp), dsb (400 bp; encoding the disulfide bond formation protein) and ftsZ (343 bp; encoding a cell division protein) genes. Sequences obtained in this study are marked in red.

PCR amplification results showed that 130 ticks were positive for *rrs*, *glt*A, and 17kD, and the prevalence of *Rickettsia* in the ticks was 20.58% (229/1113). *Rickettsia* was detected in *Haemaphysalis longicornis* (20.04%, 195/973), *Rhipicephalus microplus* (23.66%, 31/131), and *Haemaphysalis flava* (33.33%, 3/9). Table 1 shows the situation of ticks and *Rickettsia* (SFGR), *Anaplasma*, and *Ehrlichia* carried by ticks in different areas of Shaanxi Province. Based on these three genes, only one spotted fever group *Rickettsia* was detected. Among all the amplified *Rickettsia* strains, there were three representative strains (*rrs*: OR513096–OR513098; 17KD: OR526945–OR526947; *glt*A: OR526950–OR526952), and other *Rickettsia* strains were 100% identical with these three strains. As shown in the phylogenetic tree in Figure 2, and nucleotide alignment showed that their *rrs* gene, *glt*A gene, and *17*kD gene were 100, 99.89%, 99.54–100% similar to “*Candidatus* Rickettsia jingxinensis” (*Ca.* R. jingxinensis) strains (*rrs*: MH500204, MH923226; *glt*A: MH500217, OQ702260; 17kD: MH932037-MH932038, OQ702257), respectively. Figures 2–4 shows phylogenetic trees constructed with different genes, which can be used to observe the evolutionary relationships between species and to better understand the diversity of species.

### 3.3 Detection and phylogenic analysis of Anaplasma

Three *Anaplasma* species (*A. bovis, A. capra, A. marginale*) were detected in *H. longicornis* and *Rh. microplus*. *A. bovis* detected in this study was found in *H. longicornis* from Zhenba County and Baoji City with a prevalence of (3.05%, 34/1113). As shown in Figure 3, in the phylogenetic analysis based on the *rrs* gene, the 14 sequences were clustered together with those of *A. bovis* strains obtained from cattle (MH255914) and goats (MH594292, MH255920) found in other provinces of China. The topologies of *gro*EL and *glt*A gene-based phylogenetic trees were similar to that of the *rrs* gene-based phylogenetic tree. The *rrs*, *glt*A, and *gro*EL gene sequences from the strains amplified in this study shared 99.92–100%, 99.35–100%, and 99.87–100% identity with previously reported *A. bovis* sequences, respectively. *A. capra* was detected in *H. longicornis* in Baoji City and Shangluo District, with a positive rate of (3.32%, 37/1113). In the *rrs*, *glt*A, *gro*EL phylogenetic tree, the sequences obtained in this study showed a close relationship with *A. capra* isolate JZT12 (OQ185246) detected in Anhui province in China. DNA sequencing of the partial *rrs* and *glt*A gene showed 100% identity to *A. capra* isolate JZT12, while the *gro*EL gene showed 99.88–100% similarity. The positive rate of *A. marginale* was (0.18%, 2/1113), which was amplified from *H. longicornis* from Shangluo District. Sequence analysis showed that the sequences of the two samples were consistent, and were closely related to that of the known *A. marginale* found in *Rh. microplus* from wild goats and cattle (Lu et al., 2017). The sequences of their partial *rrs* gene showed 100% identity to that of the *A. marginale* strain WHBMXZ-130 (KX987367), and their *glt*A and *gro*EL gene showed 99.70 and 99.88% identity to *A. marginale* strain WHBMXZ-130, respectively.

### 3.4 Detection and phylogenic analysis of Ehrlichia

Two *Ehrlichia* species were detected in *H. longicornis* (0.27%, 3/1113). As shown in Figure 4, phylogenetic analysis of the *rrs* and *groEL* gene sequences of one detected from Zhenba revealed that it was most closely related to *E.* sp. *Yonaguni138* (HQ697588) found in Japan (Matsumoto et al., 2011). Its *rrs* and *groEL* genes were 100% identical to that of *Ehrlichia* sp. Yonaguni138. However, its *dsb* gene showed the highest identity (90.28%) to *Ca. E. pampeana* isolate S12HM25, and the *fts*Z gene showed the highest identity (90.28%) to *Ehrlichia* sp. MieHfN113. This was probably because of the absence of the counterparts of *dsb* and *fts*Z gene sequences from *Ehrlichia* sp. Yonaguni138 in the GenBank database.

**Figure 5:**
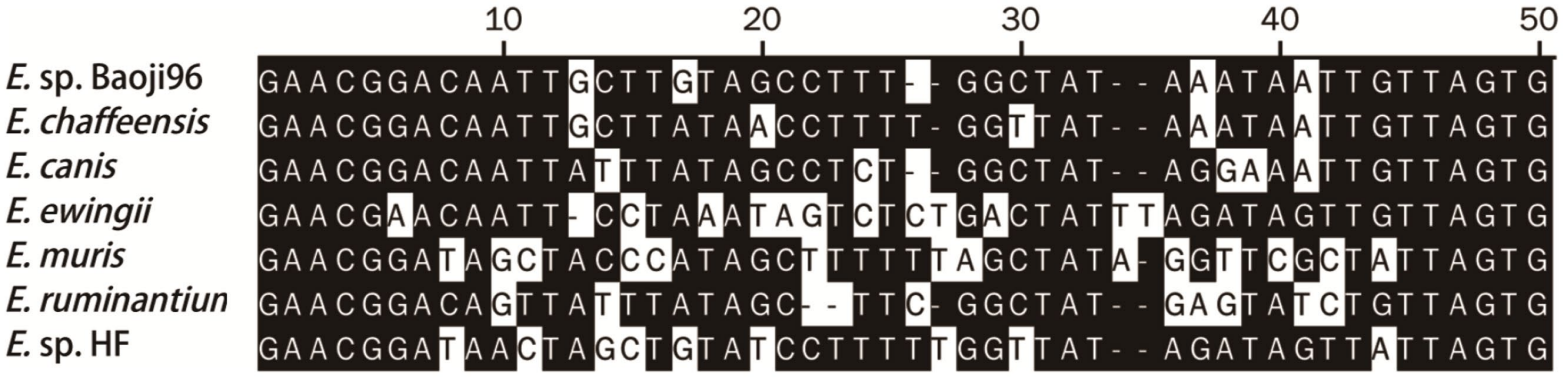
The differences in the 16S rRNA gene sequences of *E*. sp. Baoji96 and representative strains of *Ehrlichia* in a 50 bp hypervariable region located at the 5′-end of the 16S rRNA gene after multiple sequence alignment.

**Figure 6:**
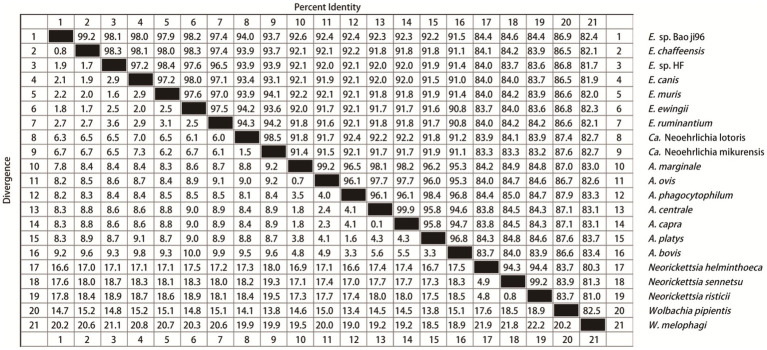
Levels of genetic identity and divergence between *E*. sp. Baoji96 and Anaplasmataceae species in the 16S rRNA gene. Twenty one sequences of representative strains of all genera of Anaplasmataceae were selected. The Clustal W algorithm was used to compare these 21 sequences in pairs and to calculate the genetic distance between them. The values on the upper right are the corrected levels of nucleotide identities for 1,390 bases.

Nucleotide alignment showed that the other *Ehrlichia* species from Baoji City was 99.20% identical to *E. chaffeensis* strain Arkansas (NR_074500) based on the *rrs* gene, 89.69% to *Ehrlichia muris* AS145 (CP006917) based on the *glt*A gene, and 94.91% to *Ehrlichia* sp. NS101 (AB454077) based on the *gro*EL gene. Thus, we believed it represented a putative novel species, which we named as “*Ehrlichia* sp. Baoji96” according to the site where it was detected. To identify Ehrlichial agents at the species level, the 5′-end and 3′-end fragments of the *rrs* gene of *Ehrlichia* sp. Baoji96 was amplified and assembled with the parts already acquired into a complete gene of 1,504 bp. The whole 16S rRNA gene sequence of this novel species was compared with those of other species of *Anaplasma* family using the Clustal W method in the multiple sequence alignment program of DNAStar. It showed that there were interspecific differences between *Ehrlichia* sp. Baoji96 and other *Ehrlichia* species in the hypervariable region at the first 200 bp of the 5′-end of the 16S rRNA gene (Figure 5). The levels of sequence divergences and similarities between the novel species and the strains of Anaplastidae are shown in Figure 6. As can be seen from the table, the entire sequence was most similar to the 16S rRNA gene sequences of *E. chaffeensis.* The similarity is 99.2%, which is less than 99.7%, and is thus in the range for a new species (Qin et al., 2019).

### 3.5 Co-infection of Rickettsiales in ticks

As is shown in Table 2, co-infection with two Rickettsiales pathogens within an individual tick was detected in 10 (0.90%, 10/1113) ticks. Five (0.45%, 5/1113) ticks were co-infected with *Ca.* R. jingxinensis and *A. bovis*, two (0.18%, 2/1113) ticks were co-infected with *Ca.* R. jingxinensis and *A. capra*, two (0.18%, 2/1113) ticks were co-infected with *Ca.* R. jingxinensis and *A. marginale*, and only one tick was co-infected with *Ca.* R. jingxinensis and *Ehrlichia* spp.

**Table 2:** Coinfection of *Rickettsia*, *Anaplasma*, and *Ehrlichia* in ticks in Shaanxi Province, China.

Ticks			Coinfection with		
Species	County	No.	*Rickettsia*	*Anaplasma*	*Ehrlichia*
*Haemaphysalis longicornis*	Zhenba	1	*Ca.* R. jingxinensis		*E.* sp. Yonaguni138
		2	*Ca.* R. jingxinensis	*A. marginale*	
		3	*Ca.* R. jingxinensis	*A. marginale*	
		4	*Ca.* R. jingxinensis	*A. bovis*	
		5	*Ca.* R. jingxinensis	*A. bovis*	
		6	*Ca.* R. jingxinensis	*A. bovis*	
	Baoji	7	*Ca.* R. jingxinensis	*A. bovis*	
		8	*Ca.* R. jingxinensis	*A. bovis*	
		9	*Ca.* R. jingxinensis	*A. capra*	
		10	*Ca.* R. jingxinensis	*A. capra*	

## 4 Discussion

In the present study, the prevalence and genetic diversity of SFGR, *Anaplasma*, and *Ehrlichia* species in Ticks (Ixodidae) in Shaanxi Province was analyzed using molecular methods. A total of 1,113 ticks, including *H. longicornis*, *Rh. microplus* and *H. flava*, were collected in Shaanxi Province from 2022 to 2023. Among these ticks, one species of *Rickettsia*, two species of *Ehrlichia*, and three species of *Anaplasma* were identified.

The *Rickettsia* species detected in this study was *Ca.* R. jingxinensis, which was first found in Japan (Ishikura et al., 2003) and named in 2016 in Jingxin city, Jilin Province, China (Liu et al., 2016). In recent years, it has been found in both *H. longicornis* and *Rh. microplus* from Liaoning, Hebei, Shaanxi, Anhui, Hubei, and Yunnan provinces of China (Dong et al., 2014; Liu et al., 2016; Wang et al., 2021b; Jin et al., 2023). In addition to *H. longicornis* and *Rh. microplus*, it was also detected in *H. flava* in Shaanxi Province in the present study. The prevalence of *Ca.* R. jingxinensis in the ticks was as high as 20.58%. And our study showed that the *glt*A genes of *Ca.* R. jingxinensis in *H. longicornis*, *Rh. microplus*, and *H. flava* were 100% identical with the *glt*A gene of *R.* sp. strain WHBMXZ-80 and *Ca.* R. longicornii, suggesting an identification of the two organisms as one species, which is consistent with previous reports (Jiang et al., 2018; Jiao et al., 2021). This suggests that *Ca.* R. jingxinensis has a much wider distribution than previously realized and might be emerging as a dominant SFG species in the epidemic distribution of the vector species. Moreover, the *glt*A gene sequence of *Ca.* R. jingxinensis was found in a patient (KU853023) (Guo et al., 2018; Guo et al., 2019a). Furthermore, we found that it can coexist with pathogenic *A. bovis* and *A. capra* in ticks. Hence, we should pay continuous attention to the agent and raise awareness of its potential pathogenicity.

*A. bovis*, *A. capra*, and *A. marginale* discovered in this study have been identified as human or animal pathogens. *A. bovis* and *A. capra* have emerged as known zoonoses in recent years, which cause considerable harm to both humans and animals (Li et al., 2015; Lu et al., 2019). In 2017, *A. bovis* was reported to be capable of infecting cattle as well as humans (Lu et al., 2019). The emergence of *A. capra* as a human pathogen was observed in Heilongjiang Province in 2015 (Li et al., 2015). Patients infected with these two pathogens have similar clinical symptoms, such as fever, chills, headache, dizziness, myalgia, rash, eschar, and lymphadenopathy (Li et al., 2015; Lu et al., 2019). A previous study showed that infections with *A. bovis* and *A. capra* in goats of Shaanxi Province were frequent in summer, perhaps because the vector ticks were more active in summer (Wang et al., 2021a). In this study, the prevalence of *A. bovis* and *A. capra* were 3.05 and 3.32%, respectively. Although there were not many *A. bovis* and *A. capra* found in this study, combined with previous studies in Shaanxi Province, *A. bovis* and *A. capra* have always existed in Shaanxi Province, suggesting that we should strengthen the investment in vector monitoring and control. Additionally, in Asia, the most important rickettsial disease for cattle is bovine anaplasmosis caused by *A. marginale* (Rodríguez et al., 2009). *A. marginale* is a pathogen belonging to the Rickettsiales, which can cause progressive anemia in ruminants, resulting in huge economic losses (Kumar et al., 2015). A study showed the presence of the disease in more than 50% of cattle sampled in tropical and subtropical regions of Mexico (Rodríguez et al., 2009). The main vectors of *A. marginale* were *Dermacentor* and *Rhipicephalus* ticks (Rodríguez et al., 2009). In this study, the DNA of *A. marginale* was detected for the first time in two *Rh. Microplus* (0.18%) collected in Zhenba County, Shaanxi Province, which proved the prevalence of *A. marginale* in Shaanxi Province. Zhenba County has both a subtropical climate and shows the presence of *A. marginale*, which reminds us that the health of cattle in this area might be facing the problem of *A. marginale* infection.

In recent decades, with the widespread use of laboratory diagnostic methods, the number of new Rickettsiales and their associated diseases has increased, and many bacteria that were previously considered non-pathogenic are now associated with human disease (Lu et al., 2017). For example, the first infection caused by *Rickettsia parkeri* was reported 70 years after it was first identified in *Amblyomma maculatum* ticks (Piotrowski and Rymaszewska, 2020). *Rickettsia slovaca* was first isolated from *Dermacentor marginatus* ticks in Czechoslovakia, several years before the first human cases were reported (Piotrowski and Rymaszewska, 2020). And, in 2021, our research group found an emerging tick-borne pathogen, named as *Ca. Ehrlichia erythraense*, which is associated with human febrile illness discovered in the Dabieshan mountain area of China. The bacteria obtained from the ticks was described as *Ehrlichia* sp. JZT12 (Lu et al., 2023). In the present study, a putative novel *Ehrlichia* species closely related to *E. chaffeensis* was first identified by gene analysis of 16S rRNA in Ixodidae from Shaanxi Province. It was named *Ehrlichia* sp. Baoji96, which showed genetic similarities for the *rrs*, *glt*A, and *gro*EL genes of 99.20% with *E. chaffeensis* strain Arkansas, 89.69% with *Ehrlichia muris* AS145, and 94.91% with *Ehrlichia* sp. NS101, respectively. In addition, the similarities of the *rrs*, *glt*A and *gro*EL genes of *Ehrlichia* sp. Baoji96 and *Ehrlichia* sp. JZT12 were 98.23, 84.24 and 91.39%, respectively. Further research is needed to determine whether this bacteria can cause disease in animals or humans.

## 5 Conclusion

In this study, we detected Ixodidae parasitized on cattle and goats in warm temperate and subtropical areas of Shaanxi Province, and analyzed the prevalence and genetic diversity of SFGR, *Anaplasma*, and *Ehrlichia* species in Ixodidae in these regions. Shaanxi Province has a diverse terrain and climate, and we have made some new discoveries: *A. marginale* was detected for the first time in *Rh. microplus* collected in Zhenba County, and a novel *Ehrlichia* species closely related to *E. chaffeensis* was first identified in *H. longicornis*. For tick-borne diseases, tick prevention is the key to avoiding infection. Hence, continuous surveillance of Rickettsiales pathogens in Chinese ticks should be conducted to assess the potential risk of transmission to animals and humans by colonizing species in disease-causing pathogens or vectors. This study provides a reference for the formulation of biological control strategies for ticks and tick-borne diseases in this area, and could improve the control effect.

## Data availability statement

The original contributions presented in the study are publicly available. This data can be found at: https://www.ncbi.nlm.nih.gov/; OR513096-OR513098, OR520945- OR520951, OR526930- OR526952.

## Ethics statement

The manuscript presents research on animals that do not require ethical approval for their study.

## Author contributions

XZ: Methodology, Writing – original draft, Writing – review & editing. WL: Methodology, Writing – original draft, Writing – review & editing. ZT: Investigation, Methodology, Writing – review & editing. NZ: Investigation, Methodology, Writing – review & editing. YZ: Investigation, Methodology, Writing – review & editing. DM: Investigation, Methodology, Writing – review & editing. LM: Investigation, Methodology, Writing – review & editing. YC: Data curation, Software, Writing – review & editing. JW: Data curation, Software, Writing – review & editing. JH: Conceptualization, Data curation, Software, Writing – review & editing. WM: Writing – review & editing. DL: Writing – original draft, Writing – review & editing. TQ: Writing – original draft, Writing – review & editing.

## Footnotes

^1^http://blast.ncbi.nlm.nih.gov/
