# Supplementary material for: Molecular detection of Rickettsiales and a potential novel Ehrlichia species closely related to Ehrlichia chaffeensis in ticks (Acari: Ixodidae) from Shaanxi Province, China, in 2022 to 2023
Source: Front Microbiol. 2024 Jan 11;14:1331434. doi: 10.3389/fmicb.2023.1331434 (PMC10808515; doi:10.3389/fmicb.2023.1331434)
Supplement: Supplementary file 1 [file Data_Sheet_1.docx]

Supplementary Material

# Supplementary Tables

## Supplementary Table 1. Primers for the amplification of sequences of ticks and tick-borne pathogens.

| **Organism** | **PCR method** | **Gene** | **Primer name** | **Sequences** | **References** |
| --- | --- | --- | --- | --- | --- |
| ticks | nested PCR | *CO* Ⅰ | TCOIF1*  TCOIR1*  TCOIF2#  TCOIR2# | GGAGCYCCWGATATAGCTTTCCC  CCTGGTAAAATTAAAATATAAACTTC  TTTTACCGCGATGAHTWTTYT  WGGRTGRCCAAARAATCAAAATA | [1] |
| Rickettsia | semi-nested PCR | *rrs* fragment 1 | R-rrs1-147F*#  R-rrs1-1175R1*  R-rrs1-1034R2# | GTACGGAATAACTTTTAGAAAT  CATGATGACTTGACRTCGT  CATCTCACGACACGAGCTG | [2] |
|  |  | *rrs* fragment 2 | R-rrs2-693F1*  R-rrs2-699F2#  R-rrs2-1427R*# | GAAGGCGRTCATYTRGGCT  GRTCATYTRGGCTRCAACTG  CTGCCTCTTGCGTTAGCT |  |
|  | semi-nested PCR | *glt*A | R-gltA-T156F2#  R-gltA-T151F1*  R-gltA-T1239R*# | CTTTATGTCTACTGCKTCTTG  CCGGGYTTTATGTCTACTGC AGCTGTCTWGGTCTGCTGATT | [2] |
|  | nested PCR | 17kD | R-r17-F1*  R-r17k-R1*  R-r17k-F2#  R-r17k-R2# | TTTACAAAATTCTAAAAACCAT  TCAATTCACAACTTGCCATT  GCTCTTGCAACTTCTATGTT  TCAATTCACAACTTGCCATT | [3] |
| Anaplasmataceae | nested PCR | *rrs* | A&E-rrs-F1*  A&E-rrs-R1*  A&E-rrs-F2#  A&E-rrs-R2# | GAA CGA ACG CTG GCG GCA AGC  AGT A(T/C)C G(A/G)A CCA GAT AGC CGC  TGC ATA GGA ATC TAC CTA GTA G  CTA GGA ATT CCG CTA TCC TCT | [4] |
| *Anaplasma* spp. | semi-nested PCR | *rrs* fragment 1 | ANArrs1-F1*#  ANArrs1-R1*  ANArrs1-R2# | GGATAGCCACTRGAARTGGTG  CGTGCTGACTTGACATCAT  CATCTCACGACACGAGCTG | [5] |
|  | semi-nested PCR | *rrs* fragment 2 | ANArrs2-F1*  ANArrs2-F2#  ANArrs2-R1*# | TGGTCCGGTACTGACRCT  TGCCTCCTTDCGGTTGGC  CTGTCTGGTCCGGTACTGAC |  |
| *A. bovis* | semi-nested PCR | *gro*EL | Ab gro-ELF1*  Ab gro-ELF2#  Ab gro-ELR*# | GTTCGCAGTATTTTGCCAGT  ATCTGGAAGRCCACTATTGAT  CTGCRTTCAGAGTCATAAATAC | [6] |
|  | semi-nested PCR | *glt*A | AB-CS-F1*  AB-CS -R*#  AB-CS-F2# | TTYATAGATGGRGATRAGGGC  AHCATTTCATRCCAYTGRG  AGATGGRGATRAGGGCATYCT | [7] |
| *A. capra* | nested PCR | *gro*EL | Ac gro-ELF1*  Ac gro-ELR3*  Ac gro-ELF2#  Ac gro-ELR2# | GCGAGGCGTTAGACAAGTCCATT  TCCAGAGATGCGAGCGTGTATAG  TGCACTGCTGGTCCAAAGGGGCT  CAACTTCGCTAGAGCCGCCAACC | [8] |
|  | semi-nested PCR | *glt*A | AC-CS-F1*  AC-CS -R*#  AC-CS-F2# | ATGATCCGGGGTTCCTGTC  TACAATACCGGAGTAAAAGT  TGCAGGTCTGAGATAACCT | [5] |
| *A. marginale* | semi-nested PCR | *gro*EL | Am gro-ELF1*  Am gro-ELR*#  Am gro-ELF2# | ACATGCTCCATACTGACTGC  AGATGCAAGCGTGTATAGCAG  AGATGAGATTGCACAGGTTG | [8] |
|  | semi-nested PCR | *glt*A | AM-CS-F1*  AM-CS -R*#  AM-CS-F2# | TGGTAGAAAAAGCGATTTTAG  CCGGTATAAAGTTGGCGT  ATAAGCTTGCCCGTTATGC | [2] |
| *Ehrlichia* spp. | semi-nested PCR | *rrs* fragment 1 | 16S-Qian-F1*#  16S-Qian-R1*  16S-Qian-R2# | TTGAGAGTTTGATCCTGGCTCAG  ATATTCCCCACTGCTGCCTC  GTTCCAGTGTGGCTGATCGT |  |
|  |  | *rrs* fragment 2 | Echaff-rrs-F1*  Echaff-rrs-R1*  Echaff-rrs-F2#  Echaff-rrs-R2# | CAAGCCTAACACATGCAAGTC  GTCACTAACCCAACCTTAAATG  ATAATTGTTAGTGGCAGACGG  AGCTTCGAGTTAAGCCAATTC | [9] |
|  |  | *rrs* fragment 3 | 16S-Hou-F1*  16S-Hou-F2#  16S-Hou-R*# | AAAAGTTGTCTCAGTTCGGA  TTGTCTCAGTTCGGATTGTT  AAAGGAGGTAATCCAGCCG |  |
|  | semi-nested PCR | *glt*A | Ehrlichia-gltA-F1*  Ehrlichia-gltA-F2*#  Ehrlichia-gltA-R* | CAGGHTTTATGTCWACTGCTGCT  TTATGTCWACTGCTGCTTGTGA  TAYAAYTGACGWGGACGACAT | [10] |
|  | semi-nested PCR | *gro*EL | Ehr-groEL-F1*  Ehr-groEL-R1*#  Ehr-groEL-F2# | TGGGCTGGYAATGAAATTGA  TCAACAGCAGCTCTAGTTG  AACATGGCAAATGTAGTTGT | [9] |
|  | semi-nested PCR | *fts*Z | Eh_ftsZ 703 R1*  Eh_ftsZ 313 F*#  Eh_ftsZ 679 R2# | CWGCTTCTCCTGTRCCCATCAT  ACTGCYGGAATGGGTGGWGGA  TTTRCCCATYTCRCTCATTATTGC | [11] |
|  | PCR | *dsb* | Eh-dsb-330 F  Eh-dsb-728 R | GATGATGTCTGAAGATATGAAACAAAT  CTGCTCGTCTATTTTACTTCTTAAAGT | [12] |

*Primers using in first round of nested PCR or semi-nested PCR

# Primers using in second round of nested PCR or semi-nested PCR

**References**

1. Duron, O.; Noël, V.; McCoy, K.D.; Bonazzi, M.; Sidi-Boumedine, K.; Morel, O.; Vavre, F.; Zenner, L.; Jourdain, E.; Durand, P.; et al. The Recent Evolution of a Maternally-Inherited Endosymbiont of Ticks Led to the Emergence of the Q Fever Pathogen, Coxiella burnetii. PLoS Pathog. 2015, 11, e1004892.

2. Guo, W.P.; Tian, J.H.; Lin, X.D.; Ni, X.B.; Chen, X.P.; Liao, Y.; Yang, S.Y.; Dumler, J.S.; Holmes, E.C.; Zhang, Y.Z. Extensive genetic diversity of Rickettsiales

bacteria in multiple mosquito species. Sci. Rep. 2016, 6, 38770.

3. Teng, Z.; Shi, Y.; Peng, Y.; Zhang, H.; Luo, X.; Lun, X.; Xia, L.; You, Y.; Li, Z.; Zhang, W.; et al. Severe Case of Rickettsiosis Identified by Metagenomic

Sequencing, China. Emerg. Infect. Dis. 2021, 27, 1530–1532.

4. Jafar Bekloo, A.; Ramzgouyan, M.R.; Shirian, S.; Faghihi, F.; Bakhshi, H.; Naseri, F.; Sedaghat, M.; Telmadarraiy, Z. Molecular Characterization and

Phylogenetic Analysis of Anaplasma spp. and Ehrlichia spp. Isolated from Various Ticks in Southeastern and Northwestern Regions of Iran. Vector Borne Zoonotic

Dis. 2018, 18, 252–257.

5. Guo, W.P.; Huang, B.; Zhao, Q.; Xu, G.; Liu, B.; Wang, Y.H.; Zhou, E.M. Human-pathogenic Anaplasma spp., and Rickettsia spp. in animals in Xi’an, China.

PLoS Negl. Trop. Dis. 2018, 12, e0006916.

6. Guo, W.P.; Wang, X.; Li, Y.N.; Xu, G.; Wang, Y.H.; Zhou, E.M. GroEL gene typing and genetic diversity of Anaplasma bovis in ticks in Shaanxi, China. Infect.

Genet. Evol. J. Mol. Epidemiol. Evol. Genet. Infect. Dis. 2019, 74, 103927.

7. Guo, W.P., Tie, W.F., Meng, S., Li, D., Wang, J.L., Du, L.Y., et al. (2020). Extensive genetic diversity of Anaplasma bovis in ruminants in Xi'an, China. Ticks

Tick Borne Dis. 11: 101477. doi:10.1016/j.ttbdis.2020.101477.

8. Remesar, S.; Prieto, A.; García-Dios, D.; López-Lorenzo, G.; Martínez-Calabuig, N.; Díaz-Cao, J.M.; Panadero, R.; López, C.M.; Fernández, G.; Díez-Baños, P.;

et al. Diversity of Anaplasma species and importance of mixed infections in roe deer from Spain. Transbound. Emerg. Dis. 2022, 69, e374–e385.

9. Teng, Z., et al., Molecular Detection of Tick-Borne Bacterial and Protozoan Pathogens in Haemaphysalis longicornis (Acari: Ixodidae) Ticks from Free-Ranging

Domestic Sheep in Hebei Province, China. Pathogens (Basel, Switzerland), 2023. 12(6).

10. Jin, X., et al., Diversity of Rickettsiales bacteria in five species of ticks collected from Jinzhai County, Anhui Province, China in 2021-2022. Frontiers In

Microbiology, 2023. 14: p. 1141217.

11. Su, H.; Onoda, E.; Tai, H.; Fujita, H.; Sakabe, S.; Azuma, K.; Akachi, S.; Oishi, S.; Abe, F.; Ando, S.; et al. Diversity unearthed by the estimated molecular

phylogeny and ecologically quantitative characteristics of uncultured Ehrlichia bacteria in Haemaphysalis ticks, Japan. Sci. Rep. 2021, 11, 687.

12. Almeida, A.P.; Souza, T.D.; Marcili, A.; Labruna, M.B. Novel Ehrlichia and Hepatozoon Agents Infecting the Crab-Eating Fox (Cerdocyon thous) in Southeastern Brazil. J. Med. Entomol. 2013, 50, 640–646.

## Supplementary Table 2. GenBank accession numbers of Rickettsiales gene sequences amplified in this study.

| **Isolate** | **16S rRNA gene** | ***glt*A gene** | ***gro*EL gene** | **17 kD gene** | ***dsb* gene** | ***fts*Z gene** |
| --- | --- | --- | --- | --- | --- | --- |
| *Ca.* R. jingxinensis SXYCA26 | OR513096 | OR526950 | . | OR526945 | . | . |
| *Ca.* R. jingxinensis SXLPA15 | OR513097 | OR526951 | . | OR526946 | . | . |
| *Ca.* R. jingxinensis SXLX76 | OR513098 | OR526952 | . | OR526947 | . | . |
| *Anaplasma marginale* SXYCA8 | OR520945 | OR526930 | OR526937 | . | . | . |
| *Anaplasma marginale* SXYCA18 | OR520946 | OR526931 | OR526938 | . | . | . |
| *Anaplasma bovis* SXYC11 | OR520947 | OR526932 | OR526939 | . | . | . |
| *Anaplasma bovis* SXLP21 | OR520948 | OR526933 | OR526940 | . | . | . |
| *Anaplasma capra* Baoji143 | OR520949 | OR526934 | OR526941 | . | . | . |
| *Anaplasma capra* Baoji142 | OR520950 | OR526935 | OR526942 | . | . | . |
| *Ehrlichia* sp. Baoji96 | OR520952 | OR526936 | OR526944 | . | . | . |
| *Ehrlichia* sp. Yonaguni138 SXZB36 | OR520951 | . | OR526943 | . | OR526948 | OR526949 |
